# Solvent-induced polymorphism in dipodal N-donor ligands containing a biphenyl core†

**DOI:** 10.1039/d3ra05713e

**Published:** 2023-10-18

**Authors:** Simran Chaudhary, Dariusz Kędziera, Zbigniew Rafiński, Liliana Dobrzańska

**Affiliations:** a Faculty of Chemistry, Nicolaus Copernicus University in Toruń Gagarina 7, 87-100 Toruń Poland lianger@umk.pl

## Abstract

Polymorph screenings for two related dipodal N-donor ligands containing a biphenyl core, namely 4,4′-bis(pyridin-4-ylmethyl)-1,1′-biphenyl (1) and 4,4′-bis(1*H*-imidazol-1-ylmethyl)-1,1′-biphenyl (2) were performed, and the new phases were isolated and their crystal structures analysed. Profiling included methods such as PXRD and thermal analysis. Hirshfeld surface analyses, as well as crystal lattice energy calculations provided deeper insight in the interplay of the intermolecular forces and the stability of the isolated phases. Furthermore, our studies revealed the presence of solvent-induced polymorphism, whereby the metastable phase is dominant upon crystallisation from THF (1a) and EtOH (2c). Upon heating, these phases transform into a more stable form, whereby the transformations were followed by PXRD studies (1, 2).

## Abstract 2

Polymorph screenings performed for two related dipodal N-donor ligands containing a biphenyl core revealed solvent-induced polymorphism.

## Graphical abstract

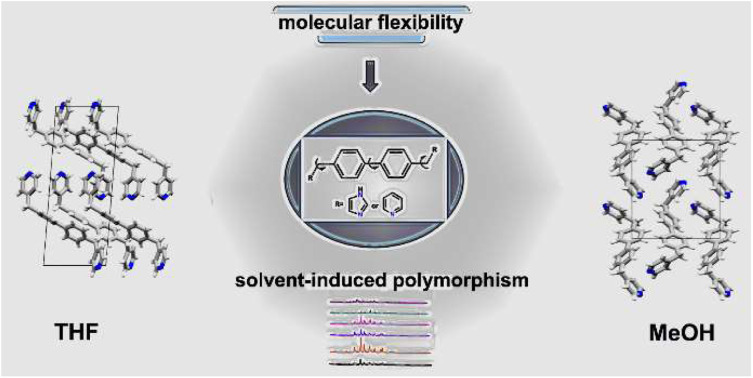

## Introduction

Polymorphism, an intriguing phenomenon concerning the formation of crystal structures^1^ that can be defined as the existence of multiple crystalline forms of the same compound,^2^ differing by molecular conformation (conformational polymorphs),^3^ molecular arrangement (packing polymorphs)^4^ or both, has been the subject of intense research in the last few decades. The existence of multiple crystalline forms of the same composition has a big impact, especially on materials science and more specifically within the pharmaceutical industry, as it makes the design of compounds of particular build and properties very challenging.^5^ It almost goes without saying that it is crucial to retain the same form of a drug in order not to be surprised by sudden changes in properties caused by the appearance of another form.^6^ It is broadly known that various synthetic/crystallisation conditions (solvent effect,^7^ the level of supersaturation,^8^ temperature^9^ and pressure^10^) can lead to the occurrence of polymorphism. The phenomenon is related to the interplay of noncovalent intermolecular forces, for example hydrogen bonds,^11^ halogen bonds^12^ and π–π interactions,^13^ different combinations of which can lead to the formation of disparate crystalline phases. Taking into account the variety of crystal structures of similar lattice energy, which can be formed, it is not trivial to predict the final product of the crystallisation process. This issue is reflected by computational Crystal Structure Prediction methods (CSP) currently being developed, which for a simple organic molecule can generate hundreds of possible polymorphs.^14^

**Scheme 1:**
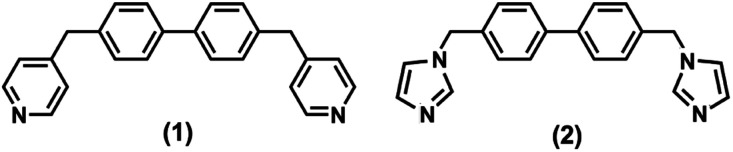
Representation of the presented N-donor ligands.

In continuation of our studies encompassing a family of dipodal N-donor ligands,^15^ we would like to present the polymorphic behaviour of two related compounds (Scheme 1) namely, 4,4′-bis(pyridin-4-ylmethyl)-1,1′-biphenyl (1) and 4,4′-bis(1*H*-imidazol-1-ylmethyl)-1,1′-biphenyl (2). Their recrystallization screenings in different solvents allowed us to isolate a series of polymorphs.

## Experimental

### Reagents and materials

All commercially available chemicals and solvents were of reagent grade and were used without further purification.

### Synthetic procedures

Both presented ligands (Scheme 1) were synthesised earlier^16^ but the procedures were modified.

#### Synthesis of 4,4′-bis(pyridin-4-ylmethyl)-1,1′-biphenyl (1)

4,4′-Bis(chloromethyl)biphenyl (2 g, 7.96 mmol), 4-pyridinylboronic acid (2.4 g, 19.52 mmol) and Na_2_CO_3_ (3.5 g, 32.51 mmol) were weighed and added in a RB flask. Under argon, Pd(PPh_3_)_4_ (0.462 g, 0.4 mmol) was added to the same flask. 1,4-Dioxane (32 ml) and water (16 ml) were added to the above flask, and the reaction mixture was refluxed at 100 °C for 4 h under argon atmosphere. Upon reaction completion (after 4 h, monitored by TLC), the mixture was cooled down, and quenched with water. The aqueous layer was extracted with DCM, and the organic layer was dried over MgSO_4_ and evaporated under reduced pressure. The resulting solid was purified by flash chromatography (eluent 0 to 3% MeOH in DCM).^17^ Yield: 13%.

^1^H NMR (CDCl_3_, 700 MHz) *δ* 8.54 (d, 4H), 7.55 (d, 4H), 7.27 (d, 4H), 7.19 (d, 4H), 4.04 (s, 4H); ^13^C NMR (CDCl_3_, 100 MHz) *δ* 149.9, 139.2, 138.0, 129.5, 127.3, 124.2, 40.9.

#### Synthesis of 4,4′-bis(1*H*-imidazol-1-ylmethyl)-1,1′-biphenyl (2)

A mixture of imidazole (3.36 g, 60 mmol) and KOH (4.08 g, 60 mmol) in 100 ml THF was stirred in a RB flask for 4 h at room temperature. Then, a solution of 4,4′-bis(chloromethyl)biphenyl in 100 ml THF was added dropwise to the above solution. After complete addition, the resulting solution was stirred for 2 days at room temperature, and then the solvent was evaporated. 50 ml of water was added to the obtained yellow solid and the aqueous layer was extracted with DCM. The organic layer was washed with water, dried with MgSO_4_ and concentrated under reduced pressure. The product was obtained as an off-white solid. Yield: 80%.

^1^H NMR (CDCl_3_, 700 MHz) *δ* 7.65 (s, 2H), *δ* 7.58 (d, 4H), *δ* 7.26 (d, 4H), 7.15 (t, 2H), 6.96 (t, 2H), 5.20 (s, 4H); ^13^C NMR (CDCl_3_, 100 MHz) *δ* 140.3, 137.4, 135.6, 129.9, 127.8, 127.6, 119.3, 50.4.

### Crystallisation of different forms of 1 and 2

The compounds 1 and 2 were recrystallized from a range of solvents of different geometry and polarity, such as acetone, acetonitrile, DCM, EtOH, MeOH and THF (10 mg of compound/10 ml of solvent). Vials covered with parafilm were left to undergo slow evaporation, which allowed us to obtain good quality crystals in all vials containing 1. In the case of 2, crystals suitable for SCXRD studies could only be grown from DCM, MeOH and EtOH (poor quality). The earlier reported crystal structures of 2 (monohydrate 2H and anhydrous form 2a) were isolated as unexpected products of the metal complexation reaction by applying slow diffusion of an ethanolic solution of AgBF_4_ into the ligand solution dissolved in chloroform or by slow diffusion of an aqueous solution of AgNO_3_ into a solution of ligand dissolved in acetone, respectively.^17^

### Measurements

^1^H and ^13^C NMR spectra were recorded on Bruker Avance 700 MHz and 400 MHz instruments, respectively and referenced to residual solvent peaks (see Fig. S1 and S2†).

Thermal analyses (TGA, DTA) were performed on a TA Instruments SDT 650 Analyser. All TGA experiments were performed at a heating rate of 2 °C min^−1^ under dry nitrogen with a flow rate of 100 ml min^−1^ covering the temperature range: 25–600 °C.

PXRD patterns were obtained on a Philips X'Pert X-ray diffractometer using CuKα radiation. The voltage and current were 40 kV and 30 mA, respectively. The samples were measured at the 2*Θ* range of 4–45° with a scan speed of 0.0089° s^−1^. All data were acquired at ambient temperature. The PXRD data were analysed using Powder Cell^18^ and Profex^19^ software.

### Structure determination

Single-crystal X-ray diffraction data for 1a, 1b, 2b and 2c were collected on an XtaLAB Synergy-S Dualflex diffractometer equipped with monochromated CuKα radiation (*λ* = 1.54184 Å). The crystals were coated with Paratone-N oil and mounted on a loop. Data collection was carried out at 100(2) K to minimize solvent loss, possible structural disorder and thermal motion effects. Data frames were processed (unit cell determination, intensity data integration, correction for Lorentz and polarisation effects, and empirical absorption correction) by using the corresponding diffractometer's software package.^20^ The structures were solved by using direct methods with SHELXS-2018/3 (ref. 21) and refined by using full-matrix least-squares methods based on *F*^2^ by using SHELXL-2018/3.^22^ The programs Mercury^23^ and POV-Ray^24^ were both used to prepare molecular graphics. All non-hydrogen atoms were refined anisotropically. All hydrogen atoms were positioned geometrically with C–H = 0.95 Å (aromatic) and 0.99 Å (methylene), and refined as riding, with *U*_iso_ (H) = 1.2 *U*_eq_ (C).

A summary of the data collection and structure refinement parameters are provided in Table 1. None of the crystal structures of 1 were reported previously, but as mentioned earlier, two forms of 2 (2H and 2a) were described before.^17^ Their unit cell parameters and basic data collection conditions are shown in Table 1. Kitaigorodskii packing indices were calculated by applying the PLATON package.^25^ The values shown for 2H and 2a are most likely underestimated and can not be directly compared to those of 2b and 2c, as the SCXRD data for the former two were collected at room temperature.

**Table 1:** Crystal data and details of the refinement parameters for the crystal structures of 1-2

Compound reference	1a	1b	2H	2a	2b	2c
Chemical formula	C_24_H_20_N_2_	C_24_H_20_N_2_	C_20_H_18_N_4_·H_2_O	C_20_H_18_N_4_	C_20_H_18_N_4_	C_20_H_18_N_4_
Formula mass	336.42	336.42	332.40	314.38	314.38	314.38
*a*/Å	20.7777(2)	5.76800(5)	4.7126(10)	10.957(1)	5.66180(10)	18.2880(5)
*b*/Å	10.58810(10)	9.98210(10)	15.269(3)	9.964(1)	14.6008(2)	7.9130(2)
*c*/Å	8.25020(10)	15.60390(10)	24.148(5)	15.457(2)	19.1251(3)	22.3941(5)
*α*/°	90	90	90	90	90	90
*β*/°	94.6050(10)	100.4410(10)	94.024(3)	94.43(1)	90.6510(10)	90
*γ*/°	90	90	90	90	90	90
Unit cell volume/Å^3^	1809.16(3)	883.546(11)	1733.33	1682.4(3)	1580.91(4)	3240.72(14)
Space group	*P*2_1_/*c*	*P*2_1_	*P*2_1_/*n*	*P*2_1_/*c*	*P*2_1_/*n*	*Pbca*
No. of formula units per unit cell, *Z*	4	2			4	8
Temperature/K	100(2)	100(2)	293(2)	293(2)	100(2)	100(2)
Radiation type	CuKα	CuKα	MoKα	MoKα	CuKα	CuKα
Absorption coefficient, *μ*/mm^−1^	0.556	0.570			0.633	0.618
No. of reflections measured	39 998	29 193			18 651	27 548
No. of independent reflections	3774	3501			3263	2985
*R*_int_	0.0347	0.0238			0.0290	0.0638
Final *R*_1_ values (*I* > 2*σ*(*I*))	0.0373	0.0256			0.0342	0.0382
Final w*R*(*F*^2^) values (*I* > 2*σ*(*I*))	0.0936	0.0674			0.0863	0.0921
Final *R*_1_ values (all data)	0.0440	0.0260			0.0384	0.0520
Final w*R*(*F*^2^) values (all data)	0.0978	0.0677			0.0892	0.0989
Goodness of fit on *F*^2^	1.028	1.038			1.052	1.033
Flack parameter		−0.13(10)				
Kitaigorodskii packing indices	68.4	70.3	68.2	66.4/66.3	71.6	69.7

### Computational methods

#### Hirshfeld surface analysis

Hirshfeld surface analysis of the polymorphs was carried out using Crystal Explorer 17.^26^ 2D fingerprint plots were generated by using a standard 0.6–2.4 Å range including reciprocal contacts.

#### Crystal lattice energy calculations

The total lattice energy, as well as contributions of its components (coulombic, polarization, dispersion and repulsion), were obtained by applying the program PIXEL.^27^ The electron densities in the crystal lattice energies were obtained on MP2/6-31G** level of theory, using the Gaussian09 quantum chemistry package.^28^

## Results and discussion

### Polymorphs of 4,4′-bis(pyridin-4-ylmethyl)-1,1′-biphenyl (1a/1b)

**Fig. 1:**
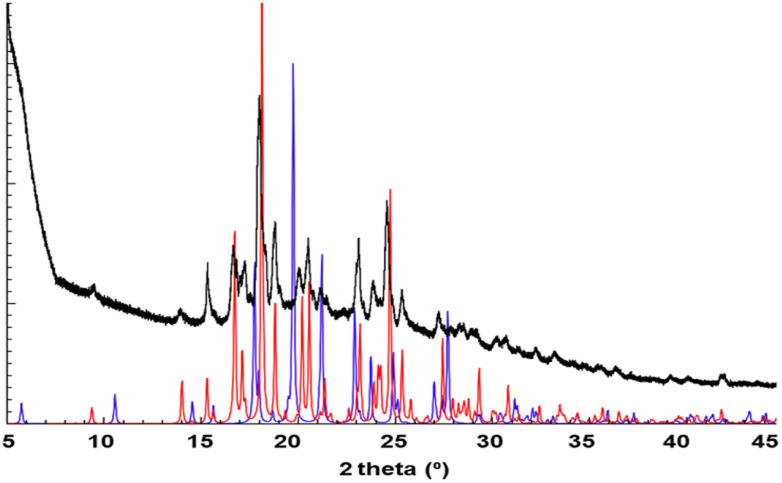
Overlay of the simulated PXRD patterns generated from crystal structures recrystallized from THF (red, 1a) and MeOH (blue, 1b), and experimental PXRD pattern obtained for the sample after recrystallization from THF (black).

PXRD screening studies performed for sample 1 recrystallized from a range of solvents indicated the formation of at least two different phases. Especially the powder pattern obtained for crystalline material grown from THF stood out (Fig. S3†), even though there was no striking difference in morphology of the crystals formed in the different solvents. We isolated single-crystals from this solvent, collected SCXRD data, determined the crystal structure (1a) and generated its powder pattern, which corresponded very well with the experimentally determined trace (Fig. 1). Furthermore, a good quality single crystal was isolated from MeOH and the crystal structure was determined (1b). Powder Cell indicated the absence of 1a in the solid recrystallized from MeOH. Moreover, the phase 1b shows its dominance in all studied solids, apart from the one obtained from THF, in which its contribution is negligible, at *ca*. 2%. Interestingly increasing the concentration of the solute in THF (15 or 20 mg/10 ml) leads to the formation of 1b exclusively.

**Fig. 2:**
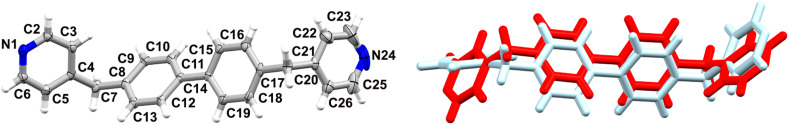
On the left: molecular structure of 1a with atomic displacement plot shown at 50% probability; the labelling refers also to 1b, on the right: overlay of 1a (red) and 1b (blue); RMSD 1.0522 Å.

The isolated polymorphs 1a and 1b crystallise in monoclinic systems of the *P*2_1_/*c* and *P*2_1_ space groups, respectively. It is worth mentioning that pairs of polymorphs crystallising in a combination of centrosymmetric and acentric space groups have previously received quite some attention, as studying these could facilitate gaining control over the formation of acentric packings.^29^ The molecules in these two crystalline forms adopt different conformations as shown in Fig. 2. The dihedral angles between the planes of the benzene rings are 34° and 35° for 1a and 1b, respectively, *versus* 57° and 20° between the planes of the pyridine rings.

The molecular packing in both crystalline phases involves sets of different intermolecular forces even though their choices in the case of this compound are rather limited (Fig. S4†). As could be expected, a large contribution is coming from C–H⋯N interactions leading to the formation of 3D supramolecular assemblies. These are further supported by C–H⋯π forces (Table 2), involving methylene groups and pyridine rings as donors and benzene rings as acceptors (1a), whereas for 1b either pyridine rings act as donors and pyridine rings as acceptors or benzene and pyridine rings as donors and benzene rings as acceptors. Furthermore, weak intermolecular π–π interactions between two adjacent pyridine rings containing N24 (symmetry operator: 1 − *x*, 1 − *y*, −*z*) are present in 1a, with a centroid–centroid distance of 3.7092(6) Å. The most striking differences between these two crystal structures lie in the strength of the interactions formed, the involvement of different molecular units in their formation, and the presence or absence of π–π interactions.

**Table 2:** Hydrogen bonding parameters for 1a and 1b^a^

Compound	D–H⋯A	H⋯A (Å)	D⋯A (Å)	D–H⋯A (°)
1a	C5–H5⋯N1^i^	2.61	3.553(2)	174
	C13–H13⋯N1^i^	2.56	3.485(2)	163
	C10–H10⋯N24^ii^	2.86	3.626(2)	139
	C16–H16⋯N24^iii^	2.74	3.617(2)	153
	C20–H20A⋯N24^iv^	2.70	3.474(2)	135
	C25–H25⋯N24^v^	2.94	3.790(2)	150
	C7–H7A⋯Cg_1_^vi^	2.85	3.767(1)	154
	C23–H23⋯Cg_1_^ii^	2.95	3.829(1)	155
1b	C7–H7B⋯N1^i^	2.73	3.521(2)	137
	C13–H13⋯N1^i^	2.74	3.585(2)	149
	C22–H22⋯N1^ii^	2.73	3.507(2)	140
	C5–H5⋯N24^iii^	2.68	3.381(2)	131
	C16–H16⋯Cg_1_^iv^	2.78	3.683(2)	158
	C2–H2⋯Cg_2_^v^	2.90	3.572(2)	129
	C23–H23⋯Cg_2_^vi^	2.89	3.680(2)	141
	C25–H25⋯Cg_1_^vii^	2.95	3.697(2)	136

a (1a) Cg_1_ is the centroid of benzene ring C14–C19; (1b) Cg_1_ is the centroid of benzene ring C8–C13, Cg_2_ is the centroid of pyridine ring N1–C6, symmetry codes (1a): (i) −*x*,1/2 + *y*,−1/2 − *z*, (ii) 1 − *x*,1 − *y*,1 − *z*, (iii) 1 − *x*,1/2 + *y*,1/2 − *z*, (iv) 1 − *x*,1 − *y*,−*z*, (v) *x*,1/2 − *y*,−1/2 + *z*, (vi) *x*, *y*, −1 + *z*; (1b): (i) −*x* + 1,*y* − 1/2,−*z* + 1, (ii) *x*,*y*,*z* + 1, (iii) −*x*,*y* + 1/2,−*z* + 2, (iv) 1 − *x*, 1/2 + *y*,2 − *z*, (v) 1 + *x*,*y*,−1 + *z*, (vi) 1 − *x*,−1/2 + *y*,2 − *z*, (vii) −*x*,−1/2 + *y*,2 − *z*.

**Fig. 3:**
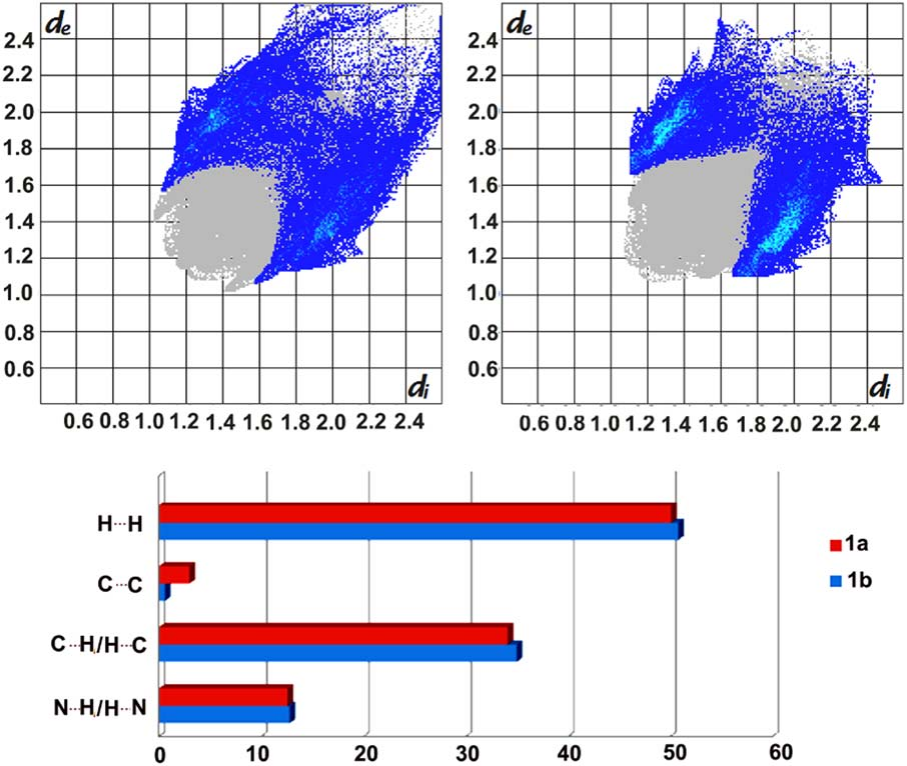
Fingerprint plots (top) for form 1a (left) and 1b (right) with the contribution of C⋯H/H⋯C contacts indicated in blue. Bottom: estimated contributions (percentages) of selected intermolecular forces stabilizing the formation of 1a and 1b.

Further analyses of the intermolecular forces stabilising the crystal structures, by applying Crystal Explorer, allowed to visualise these in the form of fingerprint plots, as well as to estimate their percentage contributions, which indicate, among others, the presence of stronger C–H⋯π interactions in 1b (Fig. 3), as observed earlier.

Moreover, the calculated enrichment ratios^30^ show once again the importance of C–H⋯N and C–H⋯π interactions in stabilising the crystal packing in 1a and 1b, with slightly higher dominance of the former in 1a (1.33 *versus* 1.15) and the latter in 1b (1.27 *vs.* 1.20).

The crystal lattice energy calculations (Table 3) indicate a lower stability of form 1a, which is in good agreement with the lower Kitaigorodskii packing index (see KI indices in Table 1). The results show once again a similar input of different forces, with the major difference in input of the dispersion term, which delivers the major contribution to stabilizing both crystalline phases.

**Table 3:** Interaction energies calculated by the program PIXEL for the two forms of 1 (kJ mol^−1^ units)

Energy component/form	Coulombic	Polarisation	Dispersion	Repulsion	Energy in total
1a	−51.0	−22.9	−198.3	94.9	−177.4
1b	−50.8	−21.9	−208.8	96.7	−184.9

To analyse the system further, thermal analyses (TG/DTA) of 1a and 1b were performed (Fig. S5 and S6†), which indicated a phase transition taking place in the case of 1a at *ca*. 100 °C. This was confirmed by a PXRD study, as heating a sample of 1a at 110 °C for 2 min revealed that this leads to irreversible conversion to 1b.

### Polymorphs of 4,4′-bis(1*H*-imidazol-1-ylmethyl)-1,1′-biphenyl (2a/2b/2c)

**Fig. 4:**
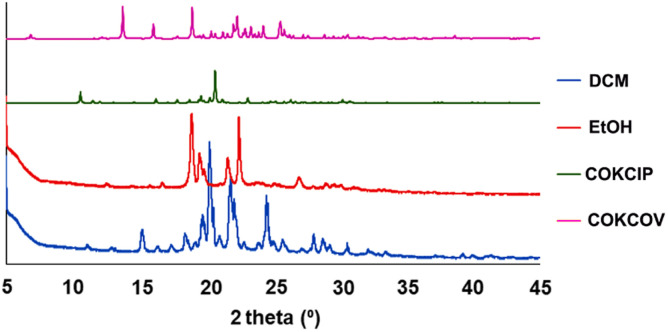
PXRD patterns obtained for samples of 2 after recrystallization from DCM (blue) and EtOH (red) and simulated PXRD patterns generated from crystal structures of 2H (COKCOV, pink) and 2a (COKCIP, green).

The crystal structure of 2 was reported and deposited at CSD earlier (refcode: COKCIP, 2a) as well as its corresponding mono-hydrated form (refcode: COKCOV, 2H). PXRD screening of solids grown from a range of solvents revealed the formation of at least two additional phases (Fig. 4). In DCM and THF, the form 2b was present exclusively, as shown by PXRD and by applying Powder Cell. Solvents such as MeOH, acetone and acetonitrile led to the formation of mixtures of 2H and 2b with a contribution of more than 70% of the latter (the highest contribution of 2b was noticed in acetone, at 92%). Interestingly, the powder pattern of crystalline material grown from EtOH allowed for isolation of another phase (2c, with a contribution of *ca.* 74%), which forms a mixture with 2H (2b was absent in this case).

**Fig. 5:**
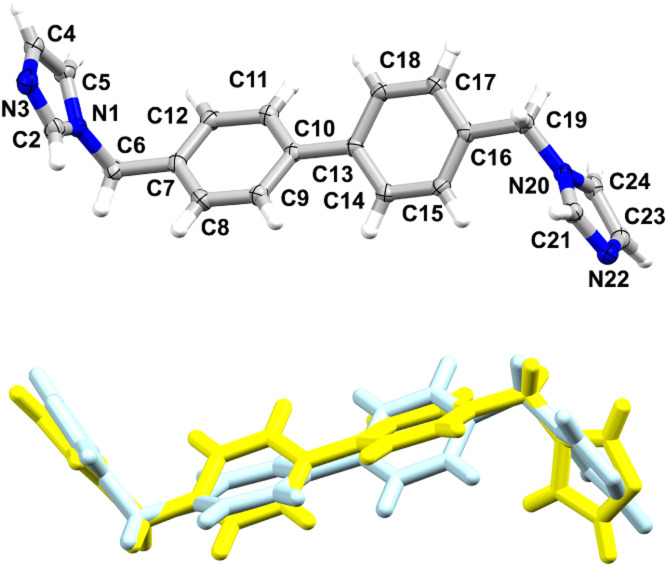
Overlay of 2b (blue) and 2c (yellow) indicating the difference in position of one of the imidazole rings (RMS deviation is 0.9791 Å).

Though the crystals of 2c were of poor quality, we managed to select a crystal suitable for SCXRD measurements (Fig. 5).

2a and 2b crystallise in monoclinic systems of the *P*2_1_/*c* and P2_1_/*n* space groups respectively, whereas 2c crystallises in the space group *Pbca* of a higher symmetry orthorhombic system. As reported earlier, one of the imidazole rings in 2a shows positional N/C disorder (50 : 50), rendering a very accurate comparison of 2a with the other two phases impossible. Comparing the conformation adopted by the ligand in 2b with the orientations adopted by the two components 2a1 and 2a2 (disorder), indicated certain differences, especially in the position of one of the imidazole rings (Fig. S7,† RMS deviation of 1.1590 Å for 2a1 and RMS deviation of 0.8922 Å for 2a2).

Another orientation of the imidazole ring is present in 2c which is facilitated by the flexibility of the molecule. The torsion angles C7–C6–N1–C2 and C16–C19–N20–C21, corresponding with the labelling in Fig. 4, are as follows: 89°/102°, 87°/−99°, 92°/−148° for 2a, 2b and 2c respectively.

The dihedral angles between the planes of the benzene rings are 33°, 31°, 34° for 2a, 2b and 2c respectively, *versus* 10°, 11° and 61° between the corresponding planes of the imidazole rings. Like in 1, the molecular packing in 2a–2c involves weak C–H⋯N hydrogen bonds, leading to the formation of 3D supramolecular assemblies supported by C–H⋯π interactions (Table 4, Fig. S8†). π–π interactions are absent in this series.

**Table 4:** Hydrogen bonding parameters for 2a–2c^a^

Compound	D–H⋯A	H⋯A (Å)	D⋯A (Å)	D–H⋯A (°)
2a1	C6–H3⋯N3^i^	2.94	3.820	158
	C10–H8⋯N3^i^	2.96	3.798	150
	C18–H15⋯N3^ii^	2.93	3.792	155
	C3–H1⋯N5^iii^	2.90	3.752	153
	C8–H5⋯N5^iv^	2.67	3.524	154
	C13–H11⋯N5^v^	2.76	3.675	168
	C4–H2⋯Cg_1_^vi^	2.69	3.575	159
	C10–H8⋯Cg_2_^vii^	2.73	3.565	150
	C12–H10⋯Cg_3_^viii^	2.95	3.677	136
2a2	C9–H6⋯N4^i^	2.95	3.598	128
	C10–H8⋯N4^ii^	2.933	3.853	173
	C3–H1⋯N5^iii^	2.90	3.752	153
	C8–H5⋯N5^iv^	2.67	3.524	154
	C13–H11⋯N5^v^	2.76	3.675	169
	C4–H2⋯Cg_1_^vi^	2.69	3.5751	159
	C10–H8⋯Cg_2_^i^	2.73	3.5650	150
	C12–H10⋯Cg_3_^iii^	2.95	3.6773	136
2b	C17–H17⋯N3^i^	2.851	3.264(2)	107
	C18–H18⋯N3^ii^	2.933	3.681(2)	137
	C19–H19B⋯N3^i^	2.733	3.318(2)	118
	C19–H19A⋯N3^iii^	2.57	3.537(2)	164
	C8–H8⋯N22^iv^	2.687	3.589(2)	154
	C6–H6B⋯N22^iv^	2.833	3.708(2)	159
	C5–H5⋯Cg_1_^v^	2.70	3.385(1)	129
	C14–H14⋯Cg_2_^vi^	2.94	3.656(1)	133
	C18–H18⋯Cg_3_^ii^	2.90	3.714(1)	144
	C21–H21⋯Cg_4_^vii^	2.90	3.835(1)	167
2c	C19–H19B⋯N3^i^	2.702	3.572(2)	147
	C21–H21⋯N3^ii^	2.852	3.629(2)	140
	C2–H2⋯N22^iii^	2.825	3.577(2)	137
	C6–H6A⋯N22^iii^	2.494	3.399(2)	152
	C6–H6B⋯N22^iv^	2.809	3.677(2)	147
	C12–H12⋯N22^iv^	2.960	3.642(2)	130
	C12–H12⋯Cg_1_^iv^	2.78	3.551(1)	139
	C19–H19A⋯Cg_2_^vi^	2.83	3.811(1)	169
	C23–H23⋯Cg_2_^vii^	2.82	3.761(1)	173

a (2a1) Cg_1_ is the centroid of the benzene ring containing C5, Cg_2_ is the centroid of the imidazole ring containing N1, Cg_3_ is the centroid of the imidazole ring containing N2; (2a2) Cg_1_ is the centroid of the benzene ring containing C5, Cg_2_ is the centroid of the imidazole ring containing N1, Cg_3_ is the centroid of the imidazole ring containing N2; (2b) Cg_1_ is the centroid of imidazole ring N20–C24, Cg_2_ is the centroid of benzene ring C7–C12, Cg_3_ is the centroid of imidazole ring N1–C5, Cg_4_ is the centroid of benzene ring C13–C18; (2c) Cg_1_ is the centroid of imidazole ring N20–C24, Cg_2_ is the centroid of benzene ring C13–C18, symmetry codes (2a1): (i) 1 − *x*,−1/2 + *y*,1/2 − *z*, (ii) 1 − *x*,1/2 + *y*,1/2 − *z*, (iii) 2 − *x*,−1/2 + *y*,−1/2 − *z*, (iv) −*x* + 2,−*x*,−*y* (v) 2 − *x*,1/2 + *y*,−1/2 − *z*, (vi) 1 − *x*,−*y*, −*z* (vii) 1 − *x*,−1/2 + *y*,1/2 − *z*, (viii) 2 − *x*,1/2 + *y*,−1/2 − *z*; (2a2): (i) 1 − *x*,1/2 + *y*,1/2 − *z*, (ii) 1 − *x*,−1/2 + *y*,1/2 − *z*, (iii) 2 − *x*,−1/2 + *y*,−1/2 − *z*, (iv) 2 − *x*,−*y*,−*z*, (v) 2 − *x*,1/2 + *y*,−*z* − 1/2, (vi) 1 − *x*,−*y*,−*z*, (vii) −*x*,−1/2 + *y*,1/2 − *z*; (2b): (i) 1/2 − *x*,1/2 + *y*,1/2 − *z*, (ii) 3/2 − *X*,1/2 + *Y*,1/2 − *Z*, (iii) −1/2 + *x*,1/2 + *y*,1/2 − *z*, (iv) −*x*,−*y*,1 − *z*, (v) 3/2 + *X*,1/2 − *Y*,−1/2 + *Z*, (vi) 1 − *X*,−*Y*,1 − *Z*, (vii) −1 + *x*,*y*,*z*; (2c): (i) *x*,1/2 − *y*,1/2 + *z*, (ii) 3/2 − *x*,1 − *y*,1/2 + *z*, (iii) *x*,3/2 − *y*,−1/2 + *z*, (iv) 2 − *x*,1 − *y*,1 − *z*, (vi) 3/2 − *x*,1/2 + *y*,*z*, (vii) 2 − *x*,2 − *y*,1 − *z*.

**Fig. 6:**
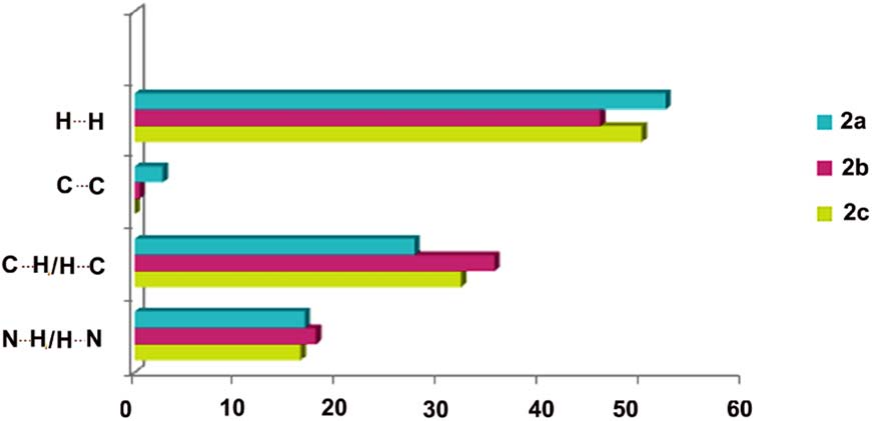
Estimated contributions (percentages) of selected intermolecular contacts to the Hirshfeld surface in 2a (blue), 2b (purple) and 2c (green).

It is worth mentioning that estimating the contributions of the different intermolecular contacts in the two separated forms of 2a, namely 2a1 and 2a2 contributing equally to molecular disorder, by applying Crystal Explorer, indicated the interplay between H⋯N (16 : 17.9%) and H⋯H (52.6 : 51.4%) forces. The results were further averaged and compared with 2b and 2c as presented in Fig. 6, indicating the largest contribution of hydrogen bonds in the case of 2b which, as shown below, is the most energetically favoured phase.

The crystal lattice energy calculation (Table 5) indicated the higher stability of form 2b over 2c, which is in good agreement with the higher Kitaigorodskii packing index of 2b (see KI indices in Table 1). It also pointed out a lower stability of phase 2a, the presence of which was not observed in any of the studied solids, and which was previously isolated in solid form after silver salt complexation. However the results for this particular phase are not very accurate, as the data set was collected at room temperature and additionally the molecule shows disorder. The lowest input of coulombic/polarisation factors observed for 2a could be the consequence of a lower input of C–H⋯π interactions, as shown on the histogram presented in Fig. 6. The results once again reveal that the dispersion term delivers the major contribution to stabilizing these three crystal phases.

**Table 5:** Interaction energies calculated by the program PIXEL for the polymorphs of 2 (kJ mol^−1^ units)

Energy component/Form	Coulombic	Polarisation	Dispersion	Repulsion	Energy in total
2a1	−43.3	−23.6	−172.1	69.3	−169.7
2a2	−56.5	−27.1	−174.2	82.5	−175.2
2a(av)^a^	−49.9	−25.35	−173.15	75.9	−172.5
2b	−72.7	−31.1	−202.8	103.9	−202.7
2c	−74.8	−29.6	−196.1	100.2	−200.3

a As this is an average of 2a disordered components, the values are (very) rough estimates.

To analyse the system further, thermal analyses (TG/DTA) of the solids obtained from DCM and EtOH were performed. These indicated a phase transition taking place at *ca*. 140 °C in the case of solid grown from EtOH (Fig. S9†). A PXRD study revealed that after heating this sample at 150 °C for 3 min, the monohydrate is completely converted to 2b, whereas the 2c phase is only partially converted. Upon extended heating at this temperature or after time (3 days in air), 2c is completely converted to 2b. Interestingly, comparing the molecular packings formed by monohydrated 2H and 2b indicates the presence of the same main packing features, which could facilitate the dehydration/hydration process. Furthermore, the results of thermal analyses pointed out much higher thermal stability of the imidazole based compounds (2) (*ca.* 30 °C) over the pyridine analogues (1).

## Conclusions

Polymorph screenings performed in a series of solvents of different geometry and polarity, such as acetone, acetonitrile, DCM, EtOH, MeOH and THF on two compounds (1 and 2) containing a biphenyl core allowed us to reveal two new phases for each.

The ability to form polymorphs is, among others, the result of the conformational flexibility of these molecules, containing aromatic rings which can rotate freely. In both cases one or more solvents could be identified, leading exclusively to the formation of the energetically more stable phase, such as MeOH in the case of 1 and DCM and THF in the case of 2. Moreover, we could also pinpoint solvents in which the metastable form was predominantly present, namely THF and EtOH, respectively, and follow the irreversible transformations of the isolated metastable forms to the stable arrangement upon heating. The presented observations show that, even in the case of similarly built compounds with a composition limiting the formation of intermolecular interactions through lack of strong hydrogen bond donors, the solvent effect on the crystallisation process can tremendously differ. Furthermore, not only the transformation of a metastable to a stable phase of different molecular packing was observed, but also the dehydration of monohydrate 2, transforming to the energetically favoured phase 2b of similar packing. Studies on related systems, as well as investigations of the solvent effect on the nucleation/crystallisation process supported by computational methods, are ongoing.

## Conflicts of interest

There are no conflicts to declare.

## Supplementary Material

RA-013-D3RA05713E-s001

RA-013-D3RA05713E-s002
