# Supplementary material for: Solvent-induced polymorphism in dipodal N-donor ligands containing a biphenyl core
Source: RSC Adv. 2023 Oct 18;13(44):30625–32. doi: 10.1039/d3ra05713e (PMC10582825; doi:10.1039/d3ra05713e)
Supplement: RA-013-D3RA05713E-s001 [file RA-013-D3RA05713E-s001.pdf]

Electronic Supplementary Material (ESI) for RSC Advances.
This journal is © The Royal Society of Chemistry 2023

Solvent-induced polymorphism in dipodal N-donor ligands containing a biphenyl core

Simran Chaudhary, Dariusz Kędziera, Zbigniew Rafiński and Liliana Dobrzańska*

Supplementary Data

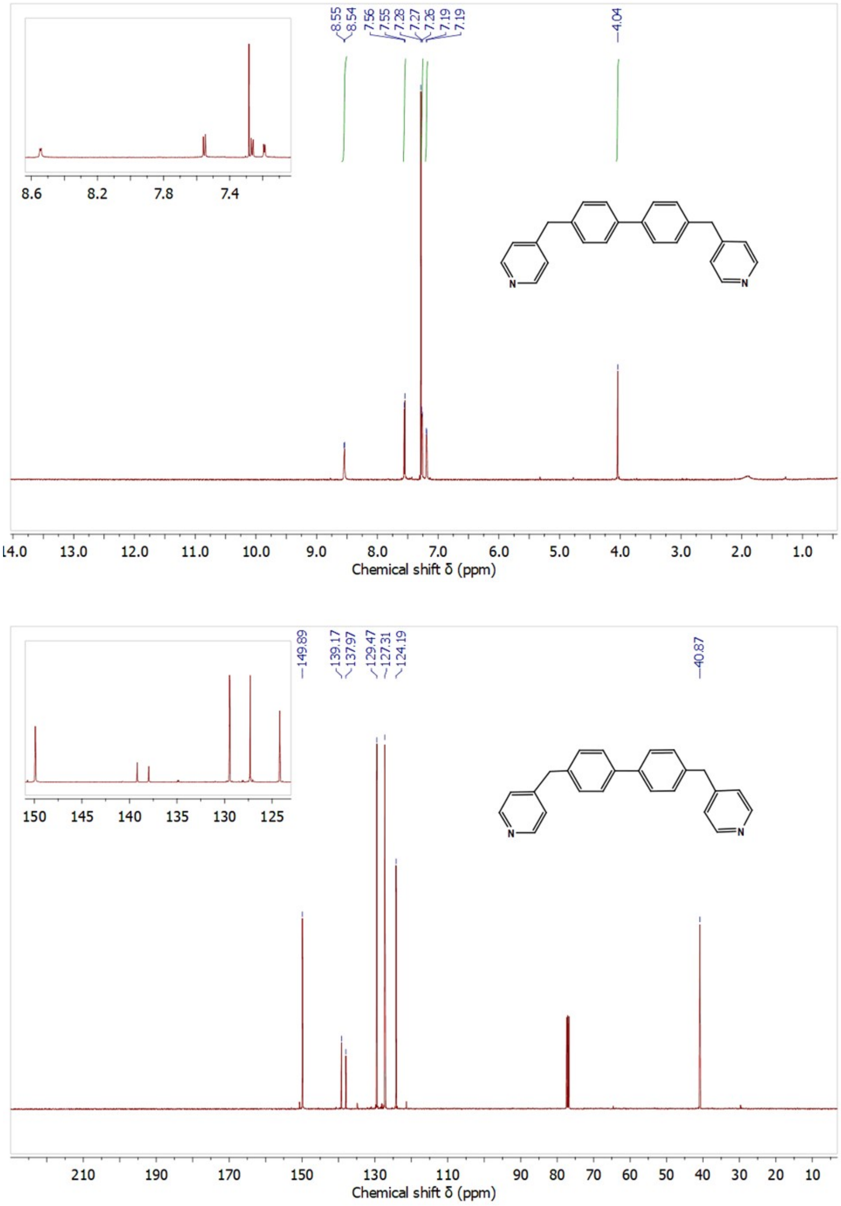

Figure S1 NMR spectra for **1**.

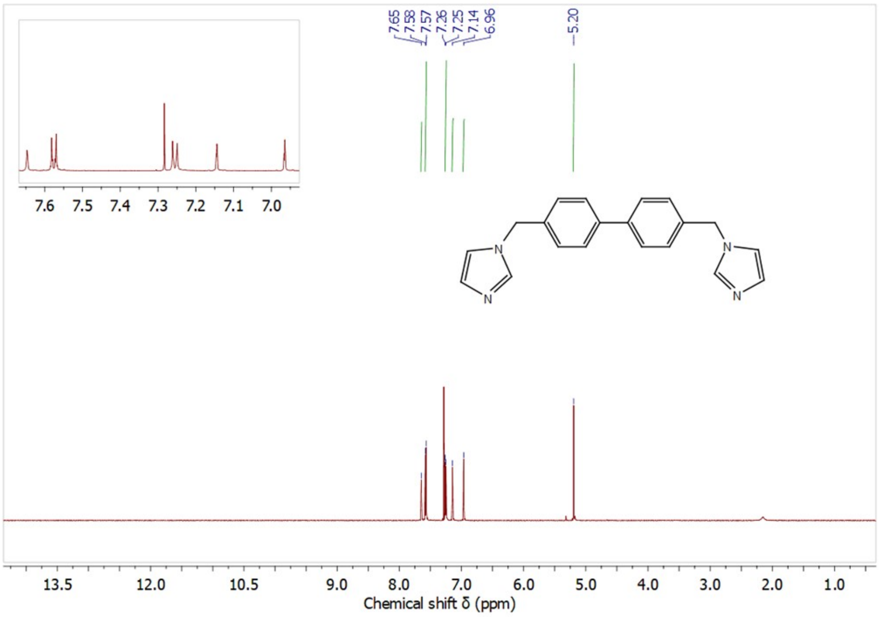

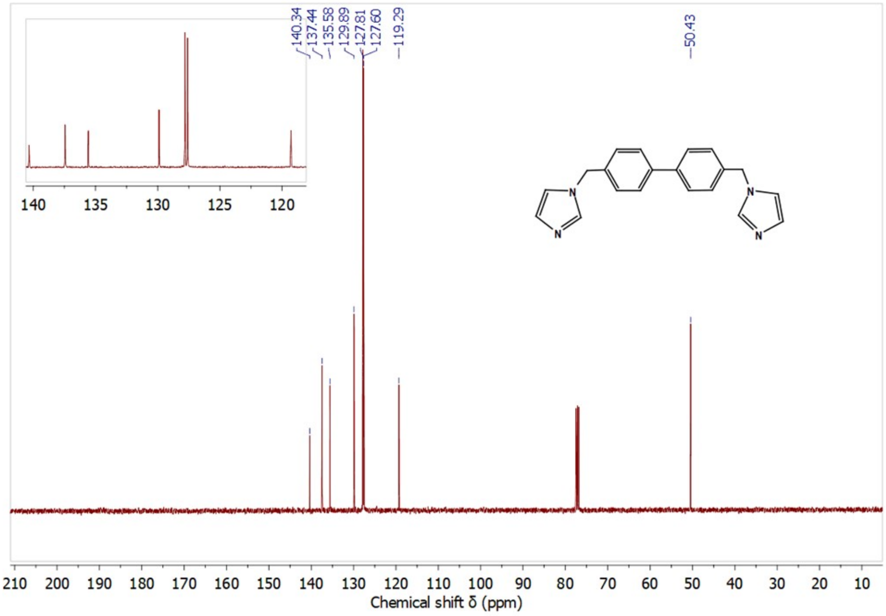

Figure S2 NMR spectra for **2**.

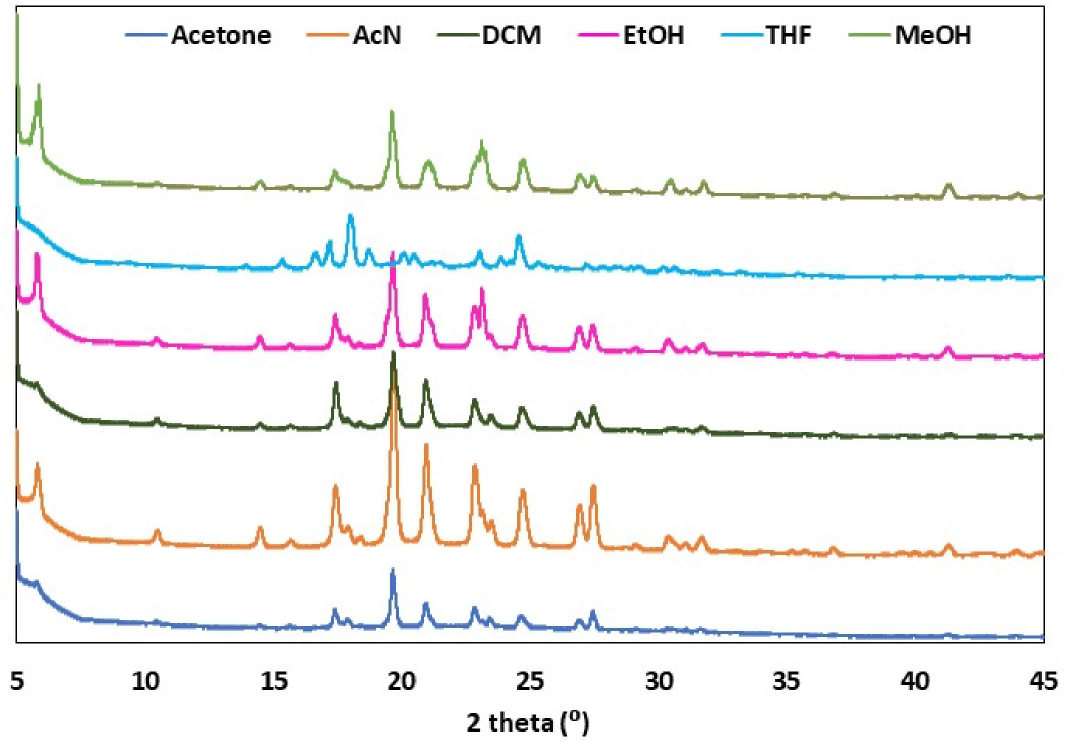

Figure S3 Powder patterns obtained for **1** upon recrystallisation from a range of solvents.

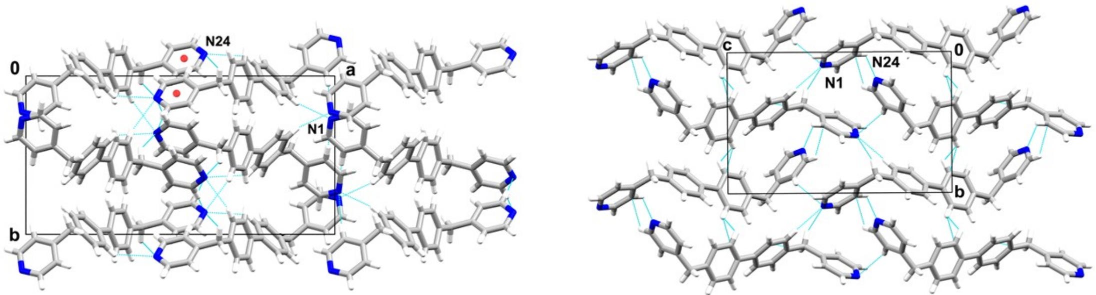

Figure S4 Packing diagrams of **1a** (on the left) and **1b** (on the right). The N-H···N interactions present are shown in blue, two centroids (in red) indicate an exemplary pair of interacting aromatic rings (π-π interactions).

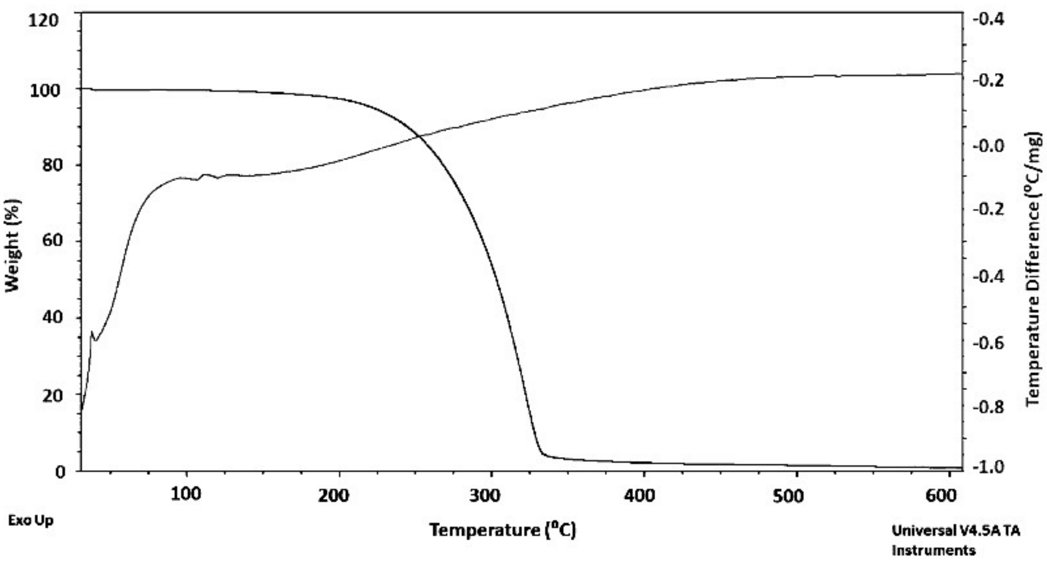

Figure S5 Thermogram of **1a**.

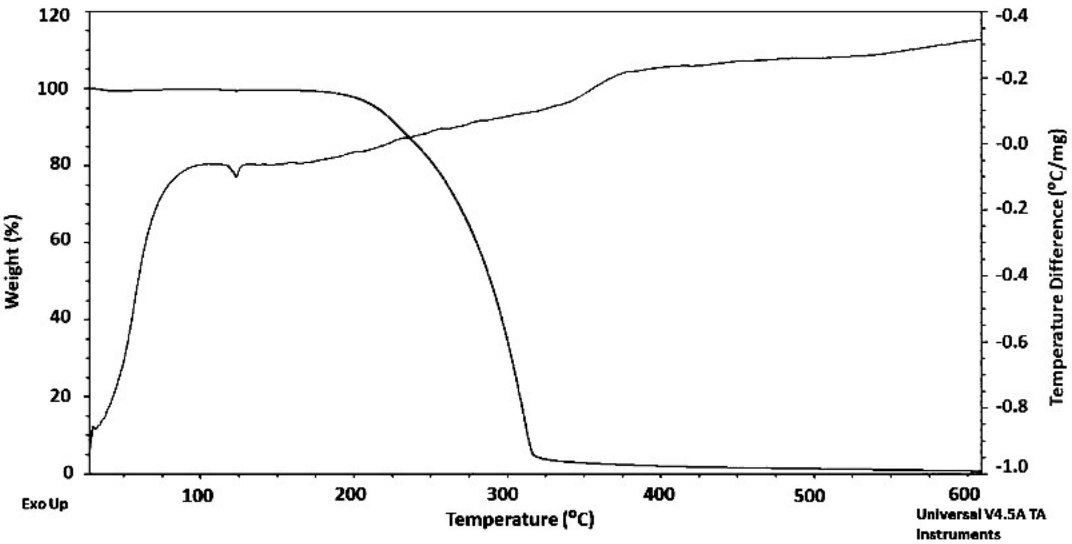

Figure S6 Thermogram of **1b**.

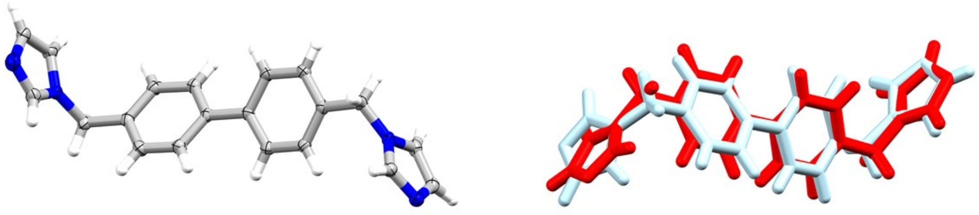

Figure S7 Molecular structure of **2b** on the left; Overlay of **2b** (blue) with one of the disordered forms of **2a (2a1)** (red) (RMSD of 1.1590).

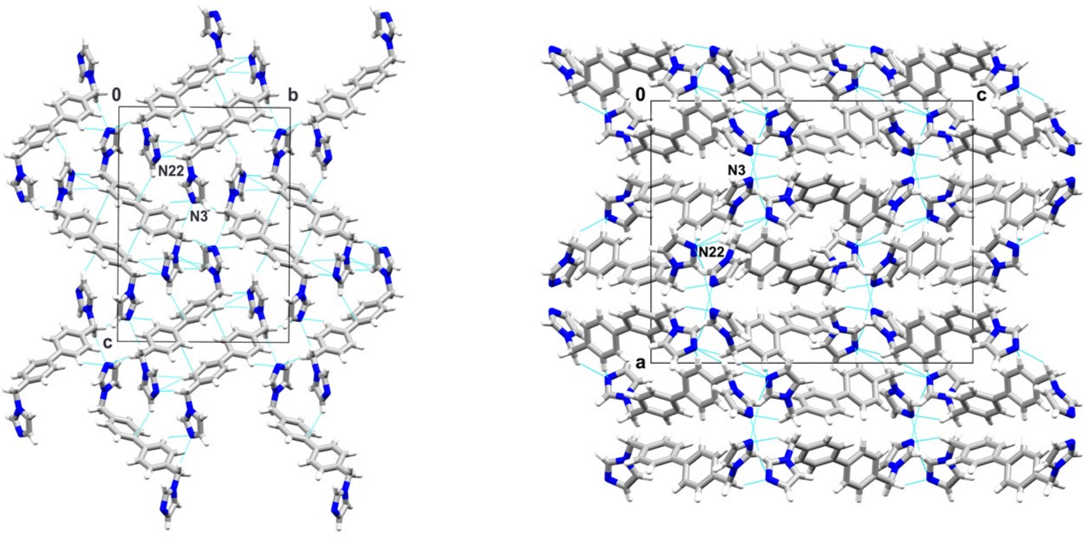

Figure S8 Packing diagrams of **2b** (on the left) and **2c** (on the right). The N-H···N interactions present are shown in blue.

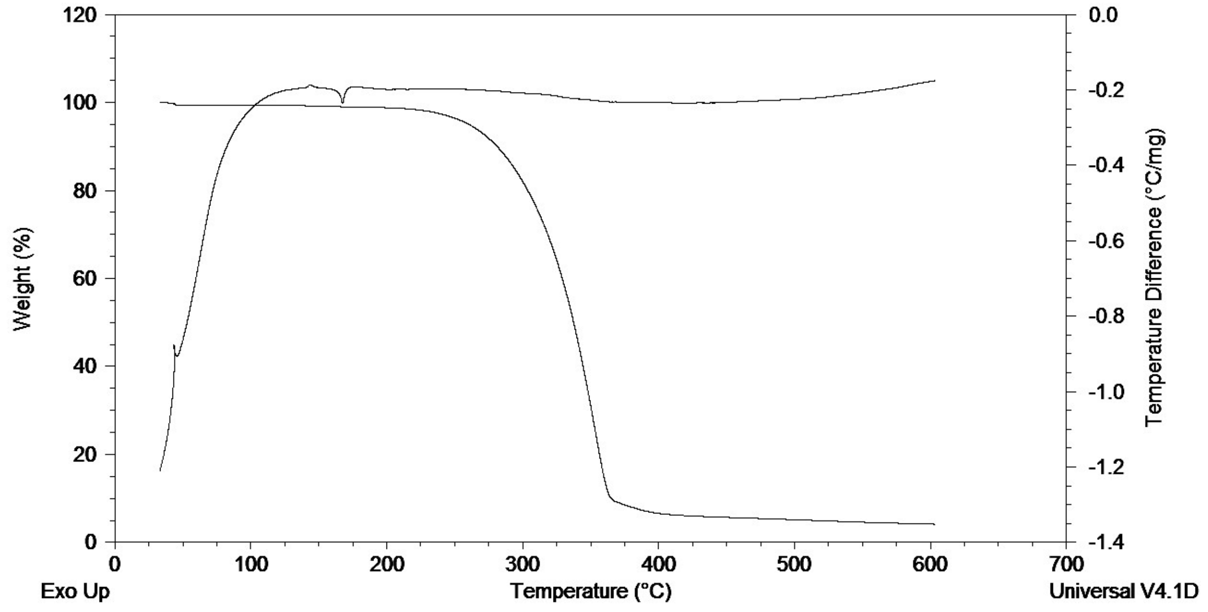

Figure S9 Thermogram of **2c**.
